# 

**Published:** 1864-08

**Authors:** 


### BOOKS AND PAMPHLETS RECEIVED.

*Man and his Relations; illustrating the influence of the Mind on the Body; the Relations of the Faculties to the Organs, and to the Elements, Objects and Phenomena of the External World.* By S. B. Brittan, M. D.

*Essays on Infant Therapeutics; to which are added Observations on Ergot; History of the origin of the use of Mercury in Inflammatory complaints; together with the Statistics of the Deaths from Poisoning in New York in the years* 1841–2–3. By John B. Beck, M. D., *Professor of Materia Medica and Medical Jurisprudence in the College of Physicians and Surgeons of the University of the State of New York; Corresponding Member of the Royal Academy of Medicine of Paris; Corresponding Member of the Medical Society of London; one of the Vice Presidents of the Academy of Medicine of New York, etc. Third edition, enlarged and revised. New York:* Wm. Wood & Co., 1864.

*The Nervous and Vascular Connection between the Mother and Fœtus in Utero.* By John O'Reilly, M. D., F. R. C. S. I., *revised and enlarged. New York,* 1864.

*Medical Communications, with the Proceedings of the Seventy-second Annual Convention of the Connecticut Medical Society, held at New Haven, May 25th and 26th,* 1864.

*Annual Announcement and Circular of Bellevue Hospital Medical College, New York,* 1864–5.

*Second Annual Announcement of the Philadelphia Dental College,* 108 *North Tenth Street, above Arch. Session of* 1864–5.

*Annual Announcement of the Departments of Medicine and Surgery, and Law, of the University of Michigan. Session of* 1864–4.

